# Automated Supraclavicular Brown Adipose Tissue Segmentation in Computed Tomography Using nnU-Net: Integration with TotalSegmentator

**DOI:** 10.3390/diagnostics14242786

**Published:** 2024-12-11

**Authors:** Kasper Jørgensen, Frederikke Engel Høi-Hansen, Ruth J. F. Loos, Christian Hinge, Flemming Littrup Andersen

**Affiliations:** 1 Department of Clinical Physiology and Nuclear Medicine, Rigshospitalet, University of Copenhagen, Blegdamsvej 9, 2100 Copenhagen, Denmark; christian.hinge@regionh.dk (C.H.); flemming.andersen@regionh.dk (F.L.A.); 2 Novo Nordisk Foundation Center for Basic Metabolic Research, Faculty of Health and Medical Sciences, University of Copenhagen, 2200 Copenhagen, Denmark; ruth.loos@sund.ku.dk; 3 Department of Clinical Medicine, University of Copenhagen, 2200 Copenhagen, Denmark

**Keywords:** brown adipose tissue, BAT segmentation, deep learning, nn-UNet, TotalSegmentator, lymphoma, PET/CT, automated segmentation

## Abstract

Background/Objectives: Brown adipose tissue (BAT) plays a crucial role in energy expenditure and thermoregulation and has thus garnered interest in the context of metabolic diseases. Segmentation in medical imaging is time-consuming and prone to inter- and intra-operator variability. This study aims to develop an automated BAT segmentation method using the nnU-Net deep learning framework, integrated into the TotalSegmentator software, and to evaluate its performance in a large cohort of patients with lymphoma. Methods: A 3D nnU-Net model was trained on the manually annotated BAT regions from 159 lymphoma patients’ CT scans, employing a 5-fold cross-validation approach. An ensemble model was created using these folds to enhance segmentation performance. The model was tested on an independent cohort of 30 patients. The evaluation metrics included the DICE score and Hausdorff Distance (HD). Additionally, the mean standardized uptake value (SUV) in the BAT regions was analyzed in 7107 FDG PET/CT lymphoma studies to identify patterns in the BAT SUVs. Results: The ensemble model achieved a state-of-the-art average DICE score of 0.780 ± 0.077 and an HD of 29.0 ± 14.6 mm in the test set, outperforming the individual fold models. Automated BAT segmentation revealed significant differences in the BAT SUVs between the sexes, with higher values in women. The morning scans showed a higher BAT SUV compared to the afternoon scans, and seasonal variations were observed, with an increased uptake during the winter. The BAT SUVs decreased with age. Conclusions: The proposed automated BAT segmentation tool demonstrates robust performance, reducing the need for manual annotation. The analysis of a large patient cohort confirms the known patterns of BAT SUVs, highlighting the method’s potential for broader clinical and research applications.

## 1. Introduction

Brown adipose tissue (BAT) is an important endocrine tissue primarily involved in energy expenditure and non-shivering thermoregulation [1,2]. Unlike white adipose tissue (WAT), which stores energy, BAT burns energy to produce heat. This function is especially crucial in newborns to maintain body temperature. The primary regions of interest for BAT are the supraclavicular and neck areas, as well as the perirenal and paravertebral regions [3].

While BAT is abundant in newborns, it is believed to regress with age. However, studies using positron emission tomography (PET) have identified metabolically active BAT in some adults, highlighting its potential role in adult metabolism [4]. The activation of BAT is mediated by the tissue-specific uncoupling protein 1 (UCP1), which uncouples oxidative phosphorylation in mitochondria, leading to an increased energy expenditure. This process has been shown to lower the plasma glucose and lipid levels in the blood, thereby improving metabolic homeostasis. Consequently, BAT has garnered significant attention for its potential therapeutic effects on various metabolic diseases, including diabetes [5,6,7,8,9]. Therefore, BAT has become an increasingly important area of research, as its unique properties open possibilities for future diagnostic opportunities and therapies, such as the transplantation or stimulation of BAT regeneration.

However, the accurate and consistent identification and quantification of BAT in imaging studies remains challenging. The manual segmentation of BAT is time-consuming, operator-dependent, and prone to variability, which motivates the use of standardized and automated segmentation methods [10]. Early efforts to segment BAT predominantly utilize simple thresholding techniques to define BAT based on a range of Hounsfield units (HUs) from computed tomography (CT) scans in combination with standardized uptake values (SUVs) from PET scans [11]. Typically, BAT is characterized as tissue with HU values ranging from −190 to −10 and exhibiting an elevated FDG uptake (SUV > 1.2) [12]. Although these methods are relatively straightforward, they are limited by the inherent subjectivity in selecting HU and SUV cutoff values [13], the latter influenced by a variety of biologic and technologic factors [14,15].

Deep learning methods have become invaluable for medical segmentation tasks, as they can directly map image data to segmentation masks without the need for handcrafted features. Among these methods, deep convolutional neural networks (CNNs) have shown exceptional performance in medical image analysis [16,17]. One of the most successful architectures is the U-Net, which has proven to be highly effective in a range of medical imaging applications, including image segmentation and synthesis [18]. Building on this, the nnU-Net extends the U-Net framework to create a fully automated and adaptive segmentation tool. Unlike traditional models, nnU-Net eliminates the need for the manual tuning of hyperparameters or architecture adjustments for each specific task. Instead, it automatically configures itself based on the dataset’s characteristics, making it highly adaptable across diverse medical imaging tasks [19]. An example of its application is TotalSegmentator, a popular open-source tool capable of segmenting over 100 different body parts from CT images. TotalSegmentator leverages nnU-Net models to perform these segmentation tasks [20]. However, most research on BAT segmentation combines imaging modalities like a PET/CT or a PET/MRI. PET imaging with a glucose tracer, such as 18F-FDG, is especially effective at highlighting metabolically active BAT, which can then be co-registered with CT anatomical data [21]. In contrast, methods that use PET for segmentation risk biasing the SUV analysis, as the segmentation depends on the very SUV activity being analyzed. While segmenting BAT from CT images alone is challenging due to the lack of specific contrast distinguishing it from other adipose tissues, CT images still provide sufficient information for BAT identification by physicians.

In this study, we propose a supraclavicular BAT segmentation model based on a 3D nnU-Net ensemble that utilizes only the anatomical information from CT images for the segmentation. The method integrates seamlessly with the existing TotalSegmentator software (version: TotalSegmentator 2.4.0), facilitating integration into the established workflows. This software already segments a wide range of anatomical regions from a CT alone, including subcutaneous and visceral fat. This suggests that BAT should also be segmentable without relying on PET data.

To demonstrate the practicality of our method, we applied it to segment a large cohort of over 7000 PET/CT scans from patients diagnosed with lymphoma. Our automated segmentation procedure efficiently generated BAT segmentation masks, which enabled the extraction of mean BAT SUVs from the corresponding PET scans. This facilitated the identification of demographic trends in the BAT SUVs with high confidence, highlighting the potential of our segmentation tool for use in BAT research and related studies.

## 2. Materials and Methods

### 2.1. Patient Cohorts

This retrospective study included 7296 whole-body FDG-PET/CT scans from 2752 patients with lymphoma undergoing staging, an interim treatment assessment, and an end-of-treatment evaluation, acquired during clinical routine at Rigshospitalet, Copenhagen, Denmark. All scans were performed between September 2017 and September 2022, and the 189 most recent scans from different patients were used for developing a segmentation model. Of these, 30 studies were kept for testing, and the remaining 159 were used for training, utilizing a 5-fold cross-validation split. The remaining 7107 scans were used for subsequent descriptive analyses of BAT. We will refer to this latter cohort as LymphBAT-7107. Patient demographics for the training and test cohorts are presented in Table 1. All patient-specific data were annomymized and managed in accordance with the Danish Data Protection Agency Act No. 502. The project was approved by the National Ethics Comité (Reference No.: 2213953).

### 2.2. PET/CT Acquisition Parameters

Whole-body (WB) CT scans, 89% of which were contrast-enhanced, were acquired using various scanners. The majority of the scans (~82%) were obtained from a Siemens Biograph 64 Vision 600 (Siemens Healthineers, Erlangen, Germany). However, multiple scans were acquired using a Biograph 128 Vision 600 Edge (~10%) and a Biograph 64 mCT Flow (~8%). Most of the scans covered the region from the mid-thigh to the top of the head, with some scans extending to include the lower extremities. Images were reconstructed with a spacing of 2.0 × 0.98 × 0.98 mm, corresponding to a median CT volume resolution of 471 × 512 × 512 voxels. All PET scans were reconstructed using point spread function (PSF) technology.

### 2.3. Manual BAT Annotation Procedure

The manual segmentation of supraclavicular BAT was performed on 189 CT images by a single reader using Mirada Medical DBx software version 01 R2 (Mirada Medical Ltd., Oxford, UK) on axial slices. The use of a single reader eliminated inter-observer variability. An experienced physician supervised the quality of the segmentations to ensure accuracy. These three-dimensional segmentation masks were used as ground truth for both the training and testing of the segmentation model.

### 2.4. Automated BAT Segmentation Using nnU-Net

The CT and BAT segmentation pairs were employed in a supervised training scheme using the nnU-Net v2 model (version: nnunetv2==2.5.1) [19], integrated within the TotalSegmentator framework (version: TotalSegmentator==2.4.0) [20]. A 5-fold cross-validation was conducted, resulting in five distinct models. Optimal preprocessing steps were automatically determined by the nnU-Net framework, utilizing the 3D full-resolution configuration. Preprocessing included intensity normalization through z-score standardization [19]. Each model was trained for 1000 epochs with a batch size of 2 using a joint DICE and cross-entropy loss function with deep supervision enabled, operating on patches of 112 × 128 × 128 voxels. Data augmentation included elastic deformations, random rotations, scaling, gamma adjustments, and intensity shifts as per the default nnU-Net settings; however, mirroring augmentations were disabled to maintain consistency with the TotalSegmentator framework settings. Model checkpoints were selected based on performance on the corresponding validation set, which may have introduced optimistic bias into the cross-validated validation metrics. This underscores the importance of the acquired independent test set.

The five models from the cross-validation were subsequently combined into an ensemble model by averaging their logits. This final ensemble model was then used for inference on the independent test set.

### 2.5. Evaluation

#### 2.5.1. BAT Segmentation Quality

The predicted BAT masks were evaluated against the ground truth BAT masks using three metrics: DICE, Intersection over Union (IoU), and Hausdorff Distance (HD) [21,22,23]. The DICE score, ranging from 0 to 1, measures the overlap between the predicted and actual BAT regions. Higher values indicate better prediction accuracy. IoU, which also ranges from 0 to 1, is defined as the ratio of the intersection to the union of the predicted and ground truth masks:

(1) $$DICE\;=\frac{2\times \left|A\cap B\right|}{\left|A\right|+\left|B\right|}=\frac{2\times TP}{2\times TP\;+FN+FP},\;IoU\;=\frac{\left|A\cap B\right|}{\left|A\cup B\right|}=\frac{TP}{TP\;+FN+FP}$$

Here, TP denotes true positive pixel predictions, FN represents false negatives, and FP indicates false positives. While DICE provides a measure of overlap, IoU focuses on the ratio of correctly predicted pixels to all pixels in the combined regions. The two metrics are complementary: DICE is particularly sensitive to small regions and balanced segmentation, while IoU emphasizes overall pixel-level accuracy. The Hausdorff Distance, ranging from 0 to infinity, measures the greatest distance from any point in a set A (predicted mask) to the closest point in another set B (ground truth mask) with smaller values indicating better prediction accuracy [21]. It is calculated as follows:

(2) $$H\left(A,B\right)=\max\left(h\left(A,B\right),h\left(B,A\right)\right),\mathrm{where}\;h\left(A,B\right)=\underset{a\in A}{\max}\underset{b\in B}{\min}{\left\|a-b\right\|}_{2}.$$

HD is particularly useful for identifying cases where false positive predictions occur far from the expected neck regions. For example, a small false positive prediction in the abdominal area will significantly affect HD but may have a negligible impact on DICE or IoU. These metrics were chosen due to their complementary strengths in evaluating segmentation accuracy. DICE and IoU assess the pixel-level overlap and accuracy, while HD captures spatial errors, providing a comprehensive evaluation of BAT segmentation performance. This combination ensures robustness in detecting both the precise alignment and outlier predictions.

#### 2.5.2. Descriptive Analyses

A statistical analysis of the standardized uptake value (SUV) signal in BAT was conducted on the LymphBAT-7107 cohort described in Section 2.1. Using the trained segmentation model, BAT masks were inferred from the CT scans, and mean BAT SUVs were extracted from the corresponding PET scans. The SUV is calculated using the following formula:

(3) $$SUV=\frac{c_{img}}{ID}\times BW.$$

In this equation, *ID* represents the injected dose in Becquerels (Bqs), *BW* refers to the patient’s body weight in kilograms, and $c_{img}$ is the activity concentration in the image measured in Bq/mL. Our goal was to compare the mean SUV in BAT across four different patients and the following study features: patient sex (M/F), patient age (Young Adults (0–39)/Middle-Aged Adults (40–59)/Older Adults (60–79)/Elderly (80+)), scan time (morning [AM]/afternoon [PM]), and season (winter/spring/summer/fall). We calculated the mean and standard error of the mean (SEM) for each subgroup and used Welch’s T-Test to determine significant general patterns in BAT SUVs across the demographic and temporal factors. We applied three thresholds for statistical significance: *p* < 0.05, *p* < 0.01, and *p* < 0.001.

## 3. Results

### 3.1. Assessment of BAT Segmentation Performance

**Figure 1:**
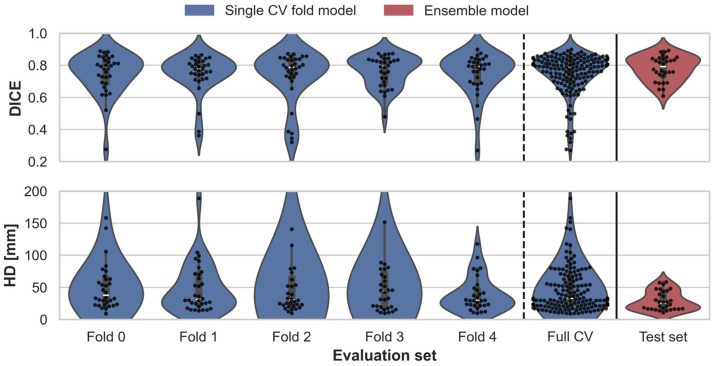
Violin plots show the distribution of DICE and HD metrics for each individual fold model in the CV, with a combined plot for full CV results. The last violin plots represent the ensemble model’s performance on the independent test set.

The evaluation metrics, DICE, IoU, and HD, for both the cross-validation (CV) models and the final ensemble model are summarized in Table 2. The DICE and HD metrics are visualized as violin plots in Figure 1. When using individual fold models, some patients obtained poor DICE and HD scores due to false positive BAT predictions far from the neck region. This issue is not observed in the combined ensemble model, which brings a drastic reduction in HD from 60.7 to 29.0 and an improvement in the DICE score from 0.749 to 0.780. The IoU also improved from 0.613 to 0.646 in the tabulated results, reflecting the enhanced pixel-level accuracy achieved by the ensemble.

**Figure 2:**
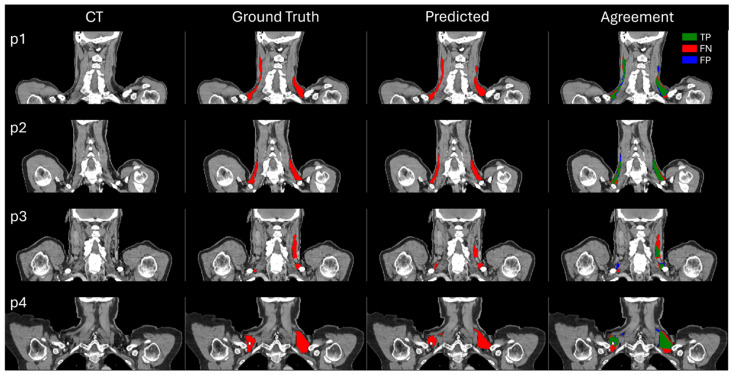
Comparison of BAT segmentation across four test patients. Each column shows the CT images, ground truth BAT annotations, model-predicted BAT segmentations, and agreement analysis showing true positive pixels (green), false negative pixels (red), and false positive pixels (blue).

Figure 2 presents a visual comparison of the model-predicted BAT segmentations against the ground truth annotations across the four representative test patients, which were randomly selected. In general, there is a strong visual correspondence between the predicted and ground truth segmentations, particularly in the regions with well-defined BAT structures. However, some inconsistencies are observed, notably in the areas where the ground truth annotations appear ambiguous or less defined. In these cases, the overlap analysis highlights the discrepancies, with true positive pixels shown in green, while the false negative (red) and false positive (blue) pixels reveal the regions where the model either missed the BAT or over-segmented, respectively.

**Figure 3:**
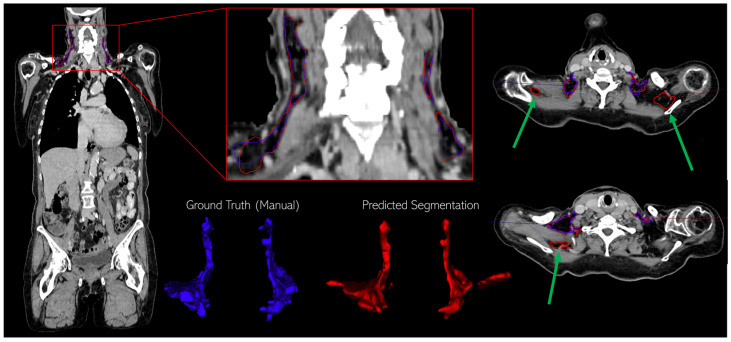
Example of manual (blue) and predicted (red) BAT regions with 3D views showing discrepancies, including unannotated predictions near the shoulders (green arrows).

Figure 3 provides a detailed example of a single representative test patient, where we display the contours of the manually annotated BAT region (blue) alongside the predicted region (red). Additionally, the segmented regions are visualized as 3D structures to better grasp the entire volume, rather than just the individual slices. For this test patient, we observed that the predicted BAT volume aligns well with the ground truth, but there are some predicted regions near the shoulders (indicated by green arrows) that were not annotated as BAT in the manual segmentation. From the 3D structure, we can see a relatively large, predicted BAT region that is disconnected from the rest of the remaining BAT volume.

### 3.2. Findings from the Descriptive Analyses in Patients with Lymphoma

**Figure 4:**
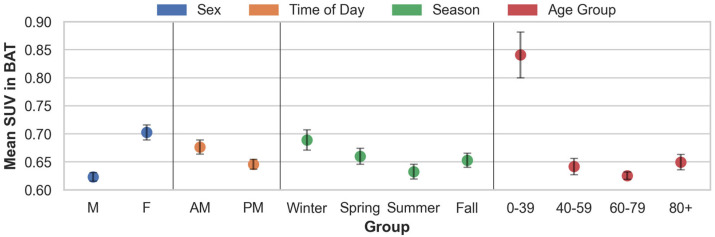
Mean values across different subject groups in the LymphBAT–7107 cohort with 95% con–fidence intervals (mean ± 1.96 SEM) shown.

The automated BAT segmentation in the LymphBAT-7107 cohort of lymphoma patients predicted an average BAT volume of 73.3 mL with a standard deviation of 47.4 mL. Figure 4 and Table 3 contain the mean SUV in BAT across the different sub-cohorts. SUV uptake is significantly higher in the females than in the males and is greater in the morning than in the afternoon. Seasonal variations are evident, with the highest uptake observed in the winter, followed by a decline in the spring, reaching its lowest point in the summer, and rising again in the fall. Additionally, the mean SUV in BAT generally decreases with age, except in the 80+ cohort, where a noticeable increase in SUV uptake is observed.

**Figure 5:**
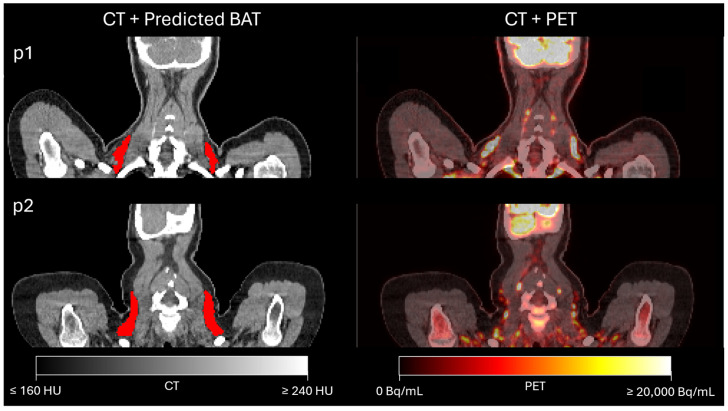
Application of the BAT segmentation model for SUV PET analysis on two test patients from the LymphBAT-7007 cohort, for which ground truth BAT annotations are unavailable. First column displays the model-inferred BAT regions, while the second shows the CT with PET overlay.

Figure 5 depicts the predicted segmentations and PET images for two example patients with metabolically active BAT. Note that the areas of increased PET activity correspond to the segmented BAT.

## 4. Discussion

This study demonstrates the feasibility and effectiveness of an automated supraclavicular brown adipose tissue (BAT) segmentation model using the nnU-Net framework, integrated within the TotalSegmentator tool. By leveraging only CT images, we have developed a robust method that bypasses the need for PET data. To the best of our knowledge, this is the first CT-only BAT segmentation model. It achieved a mean Dice score of 0.780 and a Hausdorff Distance (HD) of 29.0 mm on the independent test set, underscoring its potential for reliable and large-scale BAT segmentation.

While the reported DICE and HD scores may not seem immediately impressive compared to other segmentation tasks involving more delineated organs, which often achieve DICE scores above 0.9, it is important to consider the challenges unique to BAT. In particular, distinguishing BAT tissue from other adjacent adipose tissue is inherently difficult due to the similarity in HU values. Zhao et al. proposed BAT-Net for BAT segmentation on multi-modal magnetic resonance imaging (MRI) scans, achieving a DICE score of approximately 0.88 [24]. However, this is not directly comparable to our results, as an MRI inherently offers better differentiation between soft tissues, making the segmentation task easier. Yet, an MRI is often unavailable in many clinical settings, especially in combination with a PET scan, where PET/CT scans remain more commonly used. Another model from Wang et al., ICA-UNet which is a 2D convolutional model that takes both a CT and the corresponding PET slices as inputs, reports a DICE score of 0.91 and a HD of 7.3. However, **since** their model uses both a PET/CT and is evaluated on 2D slices and not the entire 3D volume, these metrics are also not directly comparable to our results. To address the lack of comparative studies in CT-only BAT segmentation, we propose our model as a baseline for future research.

The visual inspection of the predicted BAT regions across several test cases generally showed good correspondence with the ground truth. In the areas where inconsistencies were observed, it was often challenging to determine whether the ground truth or the predicted regions best represented the actual BAT. Some cases showed disconnected BAT areas in the shoulder regions that were absent in the ground truth annotations. This suggests that applying post-processing steps to retain only the largest BAT region on each side could potentially improve the segmentation accuracy. Despite the challenges of segmenting BAT from a CT, we consider the obtained performance sufficient for practical use, and we view our model’s simplicity and potential integration as a plug-and-play addition to TotalSegmentator as a significant advantage over the more complex multi-modal U-Net variations seen in related research. Furthermore, by excluding the PET as input we also eliminate the risk of biasing the SUV analysis.

The performance across the individual fold models was consistent, demonstrating that the training process is robust and does not heavily depend on the choice of seed or specific training data. Building on this consistency, the real performance gain comes from the ensemble model, which combines the predictions from all the fold models to mitigate the outlier predictions and significantly improve the overall segmentation metrics. However, this performance gain comes at the cost of approximately five times the inference time compared to individual models. Despite this, the ensemble inference time remains short (~1–2 min per scan), making it unlikely to pose a problem in most clinical or research workflows. Our model is freely available on https://github.com/depict-rh/bat-seg (accessed on 11 November 2024).

This study also presents, to our knowledge, the largest retrospective analysis of SUVs in BAT, providing valuable insights into the demographic and temporal factors influencing BAT SUVs. Our findings corroborate the existing literature, showing that BAT uptake was higher in women than in men [23,25]. This difference may be attributed to factors like a higher sensitivity to cold and body composition, where women typically have more subcutaneous fat, and hormonal influences, such as estrogen, and a greater sensitivity to insulin, which may enhance thermogenesis [26,27,28,29]. Additionally, women may rely more on non-shivering thermogenesis to regulate body temperature due to differences in the thermoneutral zones [30]. Secondly, the BAT SUV was higher in the individuals scanned in the morning, aligning with the research on circadian rhythms and metabolic processes [31]. The increased BAT SUVs in the morning may be driven by higher metabolic demands after waking and the fasting state, along with the cooler ambient temperatures that stimulate non-shivering thermogenesis. We also observed a higher BAT uptake during the colder months, particularly in the winter, reflecting BAT’s role in generating heat to maintain body temperature [32]. Cold exposure in the winter triggers an increased SUV uptake, consistent with the well-established link between the temperature and BAT SUV. Lastly, we found that BAT SUVs decreases with age, which is well documented [25]. This has been attributed to factors like a reduced thermogenic capacity, a decrease in BAT mass, and a reduced metabolic demand in older adults. However, an unexpected increase in BAT SUVs was observed in the elderly cohort (80+), which may be due to survivor bias, as older individuals with a higher BAT SUV likely represent a healthier subgroup [33]. These observations provide validation for our approach to quantifying BAT SUVs, as they align with the established patterns of BAT SUVs related to temperature, age, and metabolic demand.

This study has some limitations. Firstly, the automated BAT segmentation model was trained and evaluated exclusively on the CT images from lymphoma patients. While the presence of lymphoma is not expected to significantly influence BAT anatomy or physiology, which suggests the model may generalize well to other patient populations, further validation is necessary to confirm this. Additionally, the lack of PET images in the model input may limit the segmentation performance, since metabolic activity is usually a key indicator for BAT. This limitation confines our model to anatomical segmentation. However, by segmenting BAT independently of its PET activity, we avoid the risk of biasing the SUV analysis by potentially excluding the BAT regions with lower SUVs. This ensures a more objective anatomical assessment, free from metabolic influence. Furthermore, due to the time-consuming process of obtaining manual BAT segmentations, the test set was limited to 30 patients, which may be insufficient for a robust model evaluation and the identification of potential issues. Future work should include a larger test set and ground truth BAT delineations from multiple experienced clinicians. To obtain such a dataset efficiently, one could employ a human-in-the-loop approach where clinicians refine the model-predicted segmentations of new CT images [34]. Importantly, this study focused solely on the supraclavicular region for BAT segmentation, as it is a primary site for BAT deposits in adults and represents a practical starting point for developing automated methods. However, BAT can be distributed across multiple anatomical regions, such as the perirenal and paravertebral areas, which were not included in our segmentation. Future efforts could extend the model to include these regions, providing a more comprehensive BAT analysis.

Compared to the studies focused on BAT segmentation using an MRI, our study is limited to CT images. Manual BAT segmentation on CT images is inherently challenging, as certain regions can be difficult to classify as BAT. In contrast, an MRI offers better soft tissue differentiation, which may make manual segmentation on an MRI slightly more precise and potentially a more reliable gold standard for BAT segmentation. A future validation approach could involve comparing manual BAT segmentations on an MRI with the co-registered segmentations generated by our CT-based nnU-Net model. However, a PET/CT is more commonly used than a PET/MRI, which strengthens the applicability of our model. Furthermore, while currently limited to CT images, our model has the potential to serve as a foundation for transfer learning in developing a BAT segmentation model for an MRI. This would expand its applicability and contribute to the TotalSegmentator software, which also supports an MRI [35].

## 5. Conclusions

In this study, we present an automated BAT segmentation tool that can be seamlessly integrated into the TotalSegmentator software, providing a robust alternative to cumbersome manual segmentation. Our statistical analysis of the mean BAT SUV across different demographic and temporal groups of a large patient cohort identifies the key factors influencing BAT SUVs, aligning with the trends observed in the existing literature. The introduction of this segmentation tool represents a significant advancement in the standardization of a BAT analysis, facilitating more efficient investigations into BAT SUVs and promoting further research in this field.

## Figures and Tables

**Table 1:** Patient demographics (mean ± SD) for training and testing cohorts. BAT Volume refers to the manually annotated BAT volume. We do not have manually annotated BAT volumes for the LymphBAT-7107 cohort used for descriptive analysis.

Patient Cohort	Age [Years]	Weight [kg]	Height [m]	BMI	BAT Vol. [mL]
Train (*n* = 159)					
Men (*n* = 75 (47%))	63.5 ± 15.3	83.1 ± 14.4	1.80 ± 0.08	25.3 ± 4.7	117.0 ± 66.3
Women (*n* = 84 (53%))	63.4 ± 16.0	77.5 ± 16.2	1.64 ± 0.06	24.9 ± 5.4	86.0 ± 72.1
Test (*n* = 30)					
Men (*n* = 16 (53%))	57.3 ± 20.3	74.1 ± 11.7	1.79 ± 0.07	22.6 ± 3.7	97.0 ± 47.9
Women (*n* = 14 (47%))	55.3 ± 20.2	74.7 ± 21.6	1.66 ± 0.06	27.8 ± 6.8	101.4 ± 66.7
LymphBAT-7107 (*n* = 7107)					
Men (*n* = 4011 (56%))	62.2 ± 16.2	81.9 ± 15.8	1.79 ± 0.07	25.4 ± 4.5	-
Women (*n* = 3096 (44%))	63.2 ± 17.0	68.4 ± 16.0	1.66 ± 0.6	24.9 ± 5.5	-

**Table 2:** Evaluation metrics based on validation splits and the ensemble model in the independent test set. Metrics are reported as mean ± SEM.

Model	Evaluation Set	DICE↑	IoU↑	HD↓ [mm]
Single fold model	Validation Fold 0 (*n* = 32)	0.750 ± 0.022	0.613 ± 0.025	61.2 ± 13.0
	Validation Fold 1 (*n* = 32)	0.749 ± 0.021	0.611 ± 0.024	48.4 ± 6.6
	Validation Fold 2 (*n* = 32)	0.732 ± 0.028	0.598 ± 0.030	82.0 ± 24.4
	Validation Fold 3 (*n* = 32)	0.764 ± 0.017	0.627 ± 0.021	72.1 ± 20.6
	Validation Fold 4 (*n* = 31)	0.749 ± 0.023	0.614 ± 0.026	38.9 ± 4.7
	Combined Validation set (*n* = 159)	0.749 ± 0.010	0.613 ± 0.011	60.7 ± 7.2
Ensemble model	Test set (*n* = 30)	0.780 ± 0.014	0.646 ± 0.019	29.0 ± 2.7

**Table 3:** Comparison of mean SUV in BAT across different subject groups in the large LymphBAT-7107 (*n* = 7107) cohort. Statistically significant differences, *p* < 0.05, are marked with *, *p* < 0.01 are marked with **, and *p* < 0.001 are marked with ***.

Grouping	#	Mean	SEM	Welch’s *t*-Test (*p*-Values)		
Sex				F		
M	4011	0.623	0.004	*** 1.47 × 10^−23^		
F	3096	0.703	0.007	–		
Time of day				PM		
AM	2884	0.676	0.006	*** 9.44 × 10^−5^		
PM	4223	0.645	0.004	–		
Season				Spring	Summer	Fall
Winter	1734	0.689	0.009	* 0.0132	*** 8.54 × 10^−7^	* 1.44 × 10^−3^
Spring	1740	0.660	0.007	–	** 5.93 × 10^−3^	0.4880
Summer	1896	0.633	0.007	–	–	* 0.0270
Fall	1737	0.653	0.006	–	–	–
Age group				40–59	60–79	80+
0–39	876	0.841	0.021	*** 7.08 × 10^−19^	* 2.49 × 10^−23^	*** 1.18 × 10^−17^
40–59	1570	0.642	0.007	–	* 0.0448	0.4352
60–79	3938	0.625	0.003	–	–	** 2.20 × 10^−3^
80+	723	0.650	0.007	–	–	–

Note: No correction for multiple *t*-tests has been applied in this analysis.

## Data Availability

Data supporting the reported results can be obtained via contact with the corresponding author upon reasonable request and legal approval. The data are not publicly available due to no public data sharing agreement. The inference model is available on GitHub: https://github.com/depict-rh/bat-seg (accessed on 11 November 2024).
